# From the Voices of Black Dementia Caregivers: The Need for Culturally Responsive Education on Navigating Care

**DOI:** 10.1002/alz.093394

**Published:** 2025-01-09

**Authors:** Fayron Epps, Janelle Gore, Nkosi Cave, Jamiah Shoemake, Karah L Alexander, Stephanie R Bennett

**Affiliations:** ^1^ UT Health San Antonio, San Antonio, TX USA; ^2^ Emory University, Atlanta, GA USA; ^3^ MapHabit, Atlanta, GA USA

## Abstract

**Background:**

Among Black caregivers of persons living with Alzheimer’s disease and related dementias (ADRD), the overlay of health disparities and systemic discrimination substantially contribute to heightened adverse health outcomes. Black dementia caregivers report experiencing challenges in providing care that may be exacerbated by them receiving fewer support services and having more unmet needs compared to their racial counterparts. Furthermore, historical systemic failures and disadvantages impacting Black dementia caregivers contribute to long‐established stressors and vulnerabilities. Currently, there is limited caregiver education and resources that are culturally responsive to the unique needs of Black caregivers.

**Method:**

In hopes of developing an education course responding to the needs of Black dementia caregivers, focus groups were held to explore the demand and need for culturally responsive education on navigating the care of persons living with dementia. Thematic analysis was conducted on focus group sessions with current and former caregivers (N = 16).

**Result:**

Four themes emerged: Limited accessible opportunities for education and resources, Black caregivers have their own cultural identity, culturally responsive education creates a space to feel seen, and a culturally responsive course will provide a sense of empowerment. Caregivers felt that there were minimal opportunities for education and resources. However, when they were about to access resources, they were not applicable to their caregiving needs and experiences. Additionally, Black dementia caregivers acknowledged there is a cultural identity including shared experiences amongst Black caregivers. If these experiences are acknowledged within caregiver educations and resources, it would provide Black dementia caregivers with a sense of belonging, empowerment, and encouragement by showing them that they are not in this caregiving journey alone.

**Conclusion:**

These results support the development of a course that seeks to equip and empower Black dementia caregivers with the knowledge, skills, and sense of mastery they need to address and cope effectively within their role. Ultimately, a culturally responsive education course will address an urgent need to embrace the ontology of Blackness, underlying social “Blackness” and the experience of being Black in America and a dementia caregiver.

